# Supplementary material for: Molecular drivers of insecticide resistance in the Sahelo-Sudanian populations of a major malaria vector Anopheles coluzzii
Source: BMC Biol. 2023 May 24;21:125. doi: 10.1186/s12915-023-01610-5 (PMC10210336; doi:10.1186/s12915-023-01610-5)
Supplement: Supplementary file 1 — Additional file 1: Fig. S1 Sampling sites in the Sahel region of four countries. Fig. S2 Summary of total number of RNAseq reads, distribution of trimmed read length for paired reads, and unpaired read after trimming. Fig S3 Venn diagrams comparing summary of the differentially expressed genes between resistant, unexposedand susceptiblesamples. Fig. S4 Principal component analysis of the 500 most variable genes from all RNAseq experimental arms in the data from the four countries. Fig. S5 A heatmap showing the top 50 overexpressed genes in An. coluzzii populations from the Sahel region of each country. Fig. S6 Revigo Scatter and Table Views of the GO terms over-represented in all comparisonsfor data from the four countries. Fig. S7 Validation of RNA-seq results using qRT-PCR. Fig. S8 Polymorphism analysis of full-length cDNA of CYP6Z2. Fig. S9 Comparative alignment of the GSTe2 5′-UTR fragments from the various haplotypes. Fig. S10 Genetic variability of the GSTe2 5′-UTR fragments. Fig. S11 qRT-PCR validation of the overexpression of GSTe2 and CYP6Z2. [file 12915_2023_1610_MOESM1_ESM.pdf]

Additional file 1: Supplementary Figures

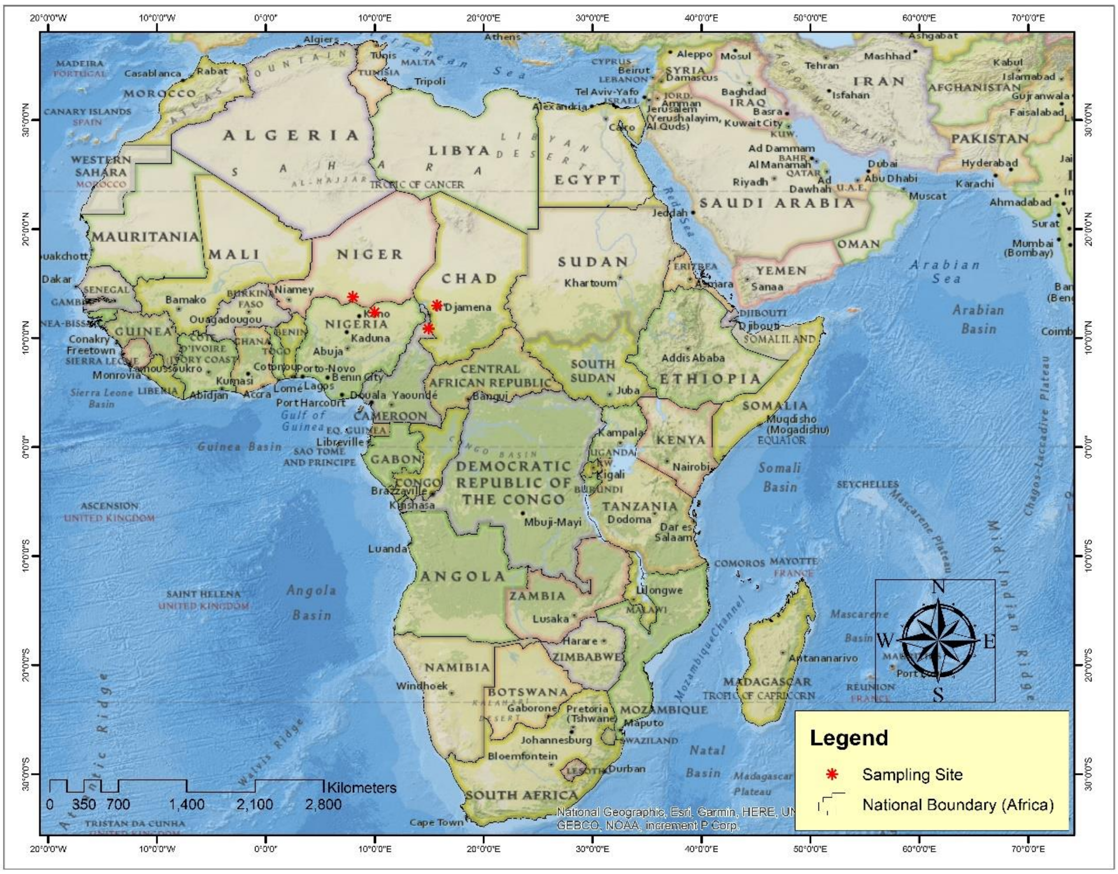

Fig. S1: Sampling sites in the Sahel region of the four countries.

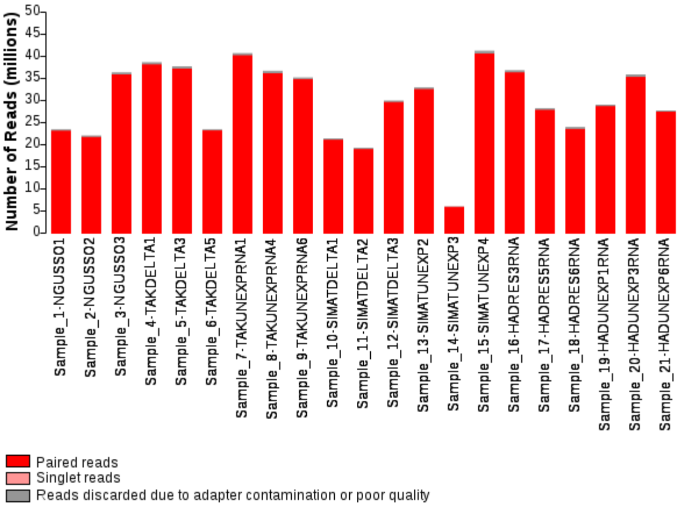

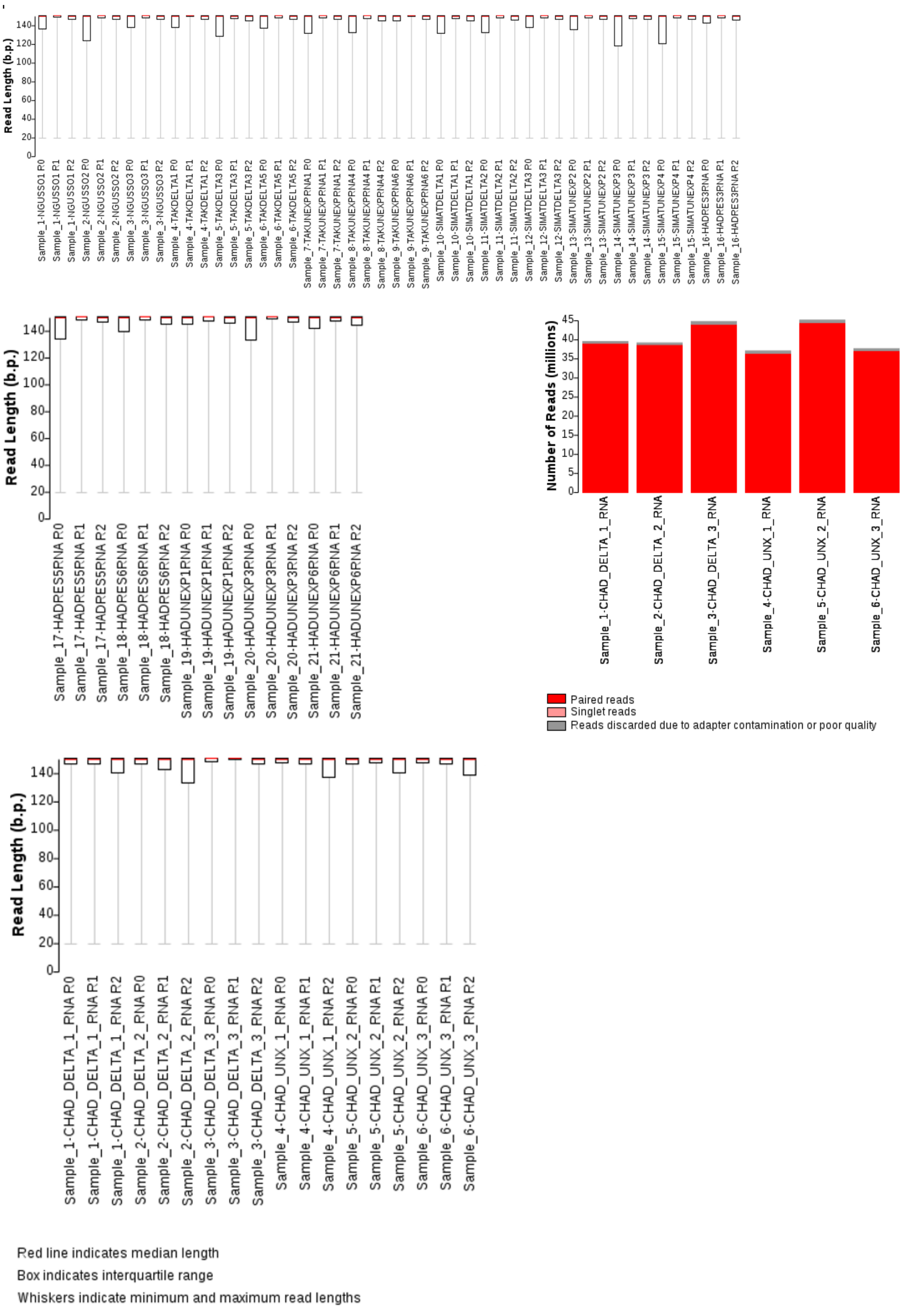

Fig. S2: Summary of total number of reads for each sample and distribution of trimmed read length for forward (R1) and reverse (R2) reads and reads unpaired after trimming (R0).

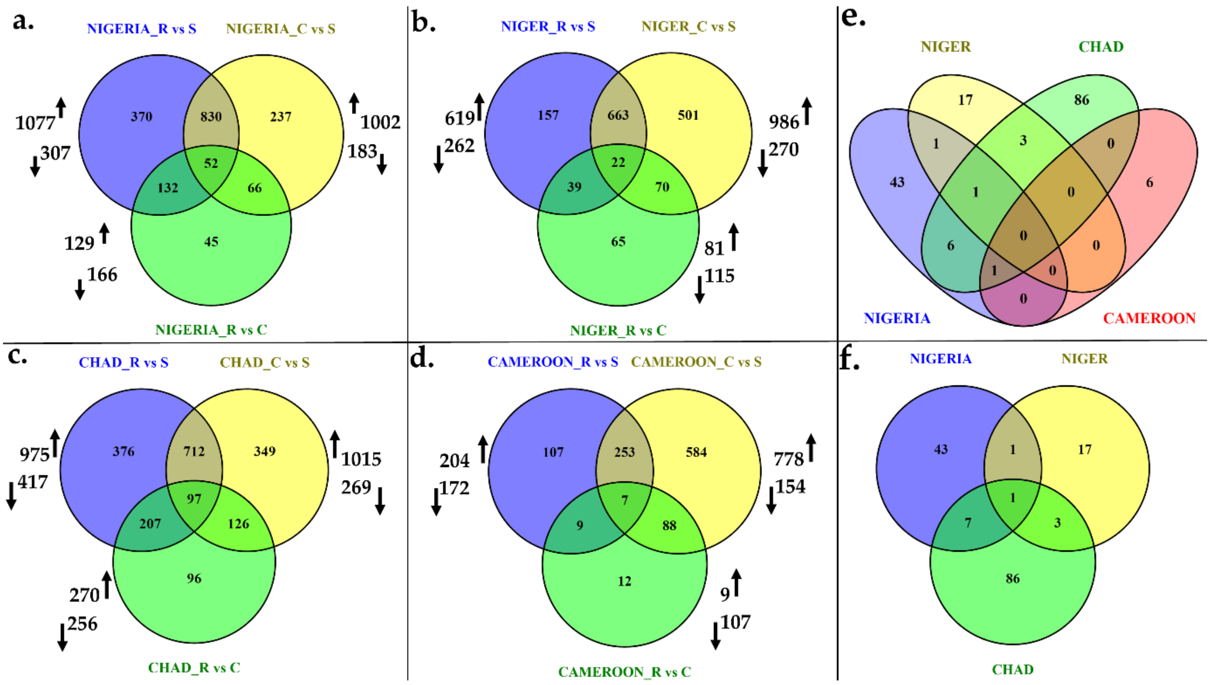

Fig. S3: Venn diagrams comparing the summary the differentially expressed (DE) genes between resistant (R), unexposed (C) and susceptible (S) samples with a transcription ratio $\log_2 FC \geq 1$ in either direction, and a corrected p value < 0.05.

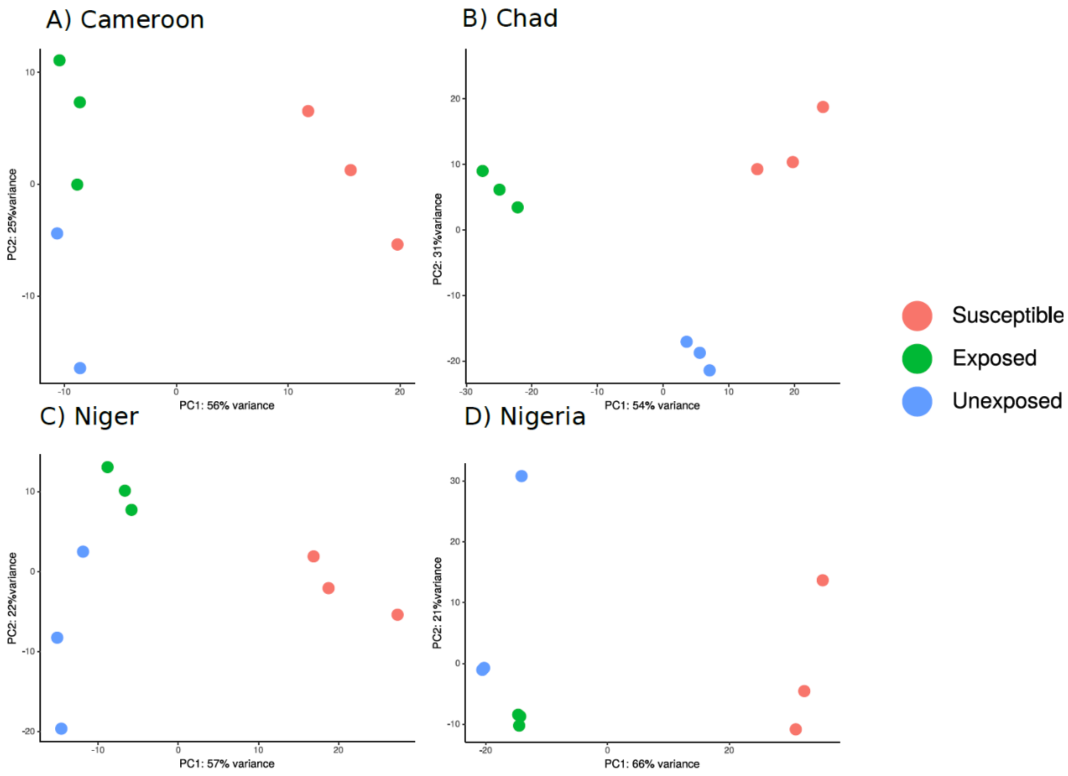

Fig. S4: Principal component analysis of the 500 most variable genes from all RNAseq experimental arms in the data from the four countries.

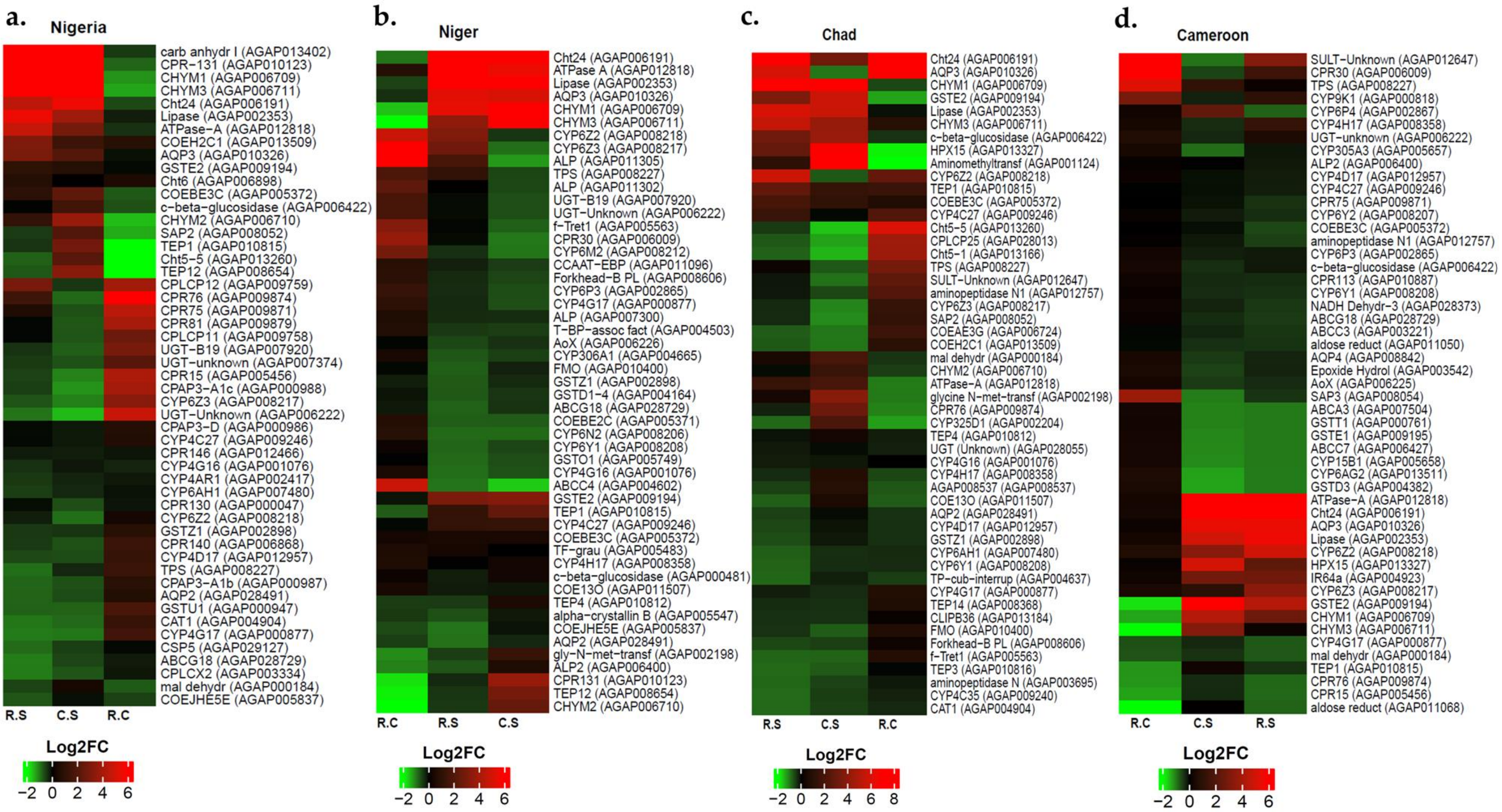

Fig. S5: A heatmap showing the top 50 overexpressed genes in *An. coluzzii* populations from the Sahel region of each country. a. for Nigeria **b.**, **c.** and **d.** for Niger, Chad and Cameroon. Several genes including chymotrypsins, CYP450s, aquaporins, glutathione S-transferases and carboxylesterases are commonly upregulated across the Sahel of the four countries.

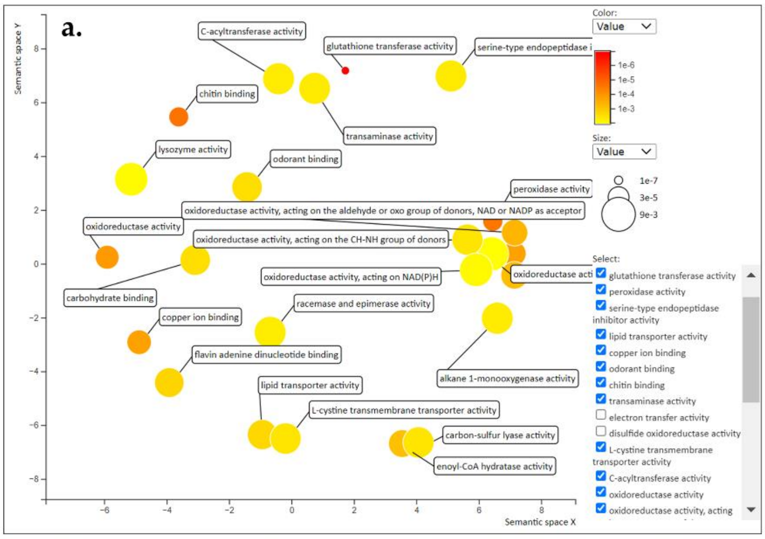

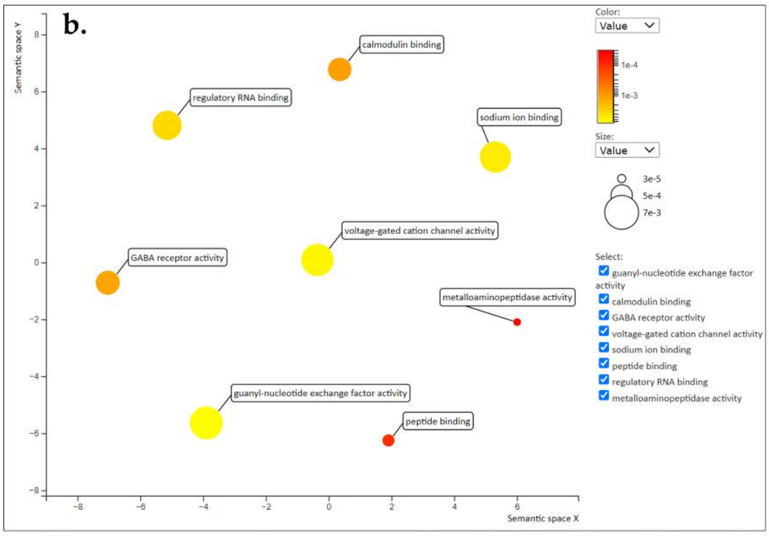

Term ID	Name	Frequency	Pin?	Value	Uniqueness	Dispensability
GO:0004867	serine-type endopeptidase inhibitor activity	0.121%		0.0039	0.91	0.00
GO:0015184	L-cystine transmembrane transporter activity	0.001%		0.0030	0.98	0.00
GO:0016616	oxidoreductase activity, acting on the CH-OH group of donors, NAD or NADP as acceptor	1.855%		0.0095	0.80	0.00
GO:0030246	carbohydrate binding	0.797%		0.0023	0.98	0.00
GO:0003796	lysozyme activity	0.043%		0.0078	0.93	0.03
GO:0016408	C-acyltransferase activity	0.080%		0.0040	0.95	0.03
GO:0016846	carbon-sulfur lyase activity	0.134%		0.0030	0.94	0.03
GO:0016854	racemase and epimerase activity	0.375%		0.0042	0.93	0.03
GO:0005549	odorant binding	0.051%		0.0023	0.99	0.04
GO:0008061	chitin binding	0.116%		0.0000	0.98	0.05
GO:0016491	oxidoreductase activity	12.887%		0.0001	0.95	0.05
GO:0050660	flavin adenine dinucleotide binding	1.631%		0.0014	0.97	0.06
GO:0005507	copper ion binding	0.292%		0.0002	0.98	0.14
GO:0008483	transaminase activity	0.728%		0.0039	0.94	0.20
GO:0004364	glutathione transferase activity	0.086%		0.0000	0.95	0.20
GO:0018685	alkane 1-monooxygenase activity	0.001%		0.0040	0.88	0.23
GO:0005319	lipid transporter activity	0.146%		0.0017	0.98	0.30
GO:0015036	disulfide oxidoreductase activity	0.326%		0.0005	0.83	0.37
GO:0016645	oxidoreductase activity, acting on the CH-NH group of donors	0.350%		0.0030	0.83	0.37
GO:0004601	peroxidase activity	0.427%		0.0000	0.77	0.38

Term ID	Name	Frequency	Pin?	Value	Uniqueness	Dispensability
GO:0005085	guanyl-nucleotide exchange factor activity	0.309%		0.0070	0.90	0.00
GO:0016917	GABA receptor activity	0.027%		0.0011	0.95	0.00
GO:0022843	voltage-gated cation channel activity	0.155%		0.0060	0.43	0.00
GO:0031402	sodium ion binding	0.009%		0.0048	0.91	0.00
GO:0070006	metalloaminopeptidase activity	0.153%		0.0000	1.00	0.00
GO:0061980	regulatory RNA binding	0.013%		0.0031	0.99	0.03
GO:0005516	calmodulin binding	0.135%		0.0009	0.99	0.03
GO:0042277	peptide binding	0.100%		0.0001	0.99	0.04
GO:0008273	calcium, potassium:sodium antiporter activity	0.006%		0.0048	0.49	0.47
GO:0046961	proton-transporting ATPase activity, rotational mechanism	0.115%		0.0023	0.53	0.57
GO:0099095	ligand-gated anion channel activity	0.006%		0.0008	0.49	0.64
GO:0022848	acetylcholine-gated cation-selective channel activity	0.024%		0.0000	0.43	0.70
GO:0005283	amino acid:sodium symporter activity	0.045%		0.0031	0.45	0.72
GO:0005328	neurotransmitter:sodium symporter activity	0.001%		0.0000	0.58	0.73
GO:0008028	monocarboxylic acid transmembrane transporter activity	0.052%		0.0013	0.54	0.74
GO:0022839	ion gated channel activity	0.054%		0.0000	0.53	0.74
GO:0005254	chloride channel activity	0.123%		0.0000	0.45	0.80
GO:0005245	voltage-gated calcium channel activity	0.031%		0.0011	0.44	0.81
GO:0015171	amino acid transmembrane transporter activity	0.268%		0.0000	0.59	0.83
GO:0030955	potassium ion binding	0.068%		0.0048	0.91	0.86
GO:0005096	GTPase activator activity	0.247%		0.0003	0.90	0.87
GO:0005249	voltage-gated potassium channel activity	0.119%		0.0022	0.41	0.90

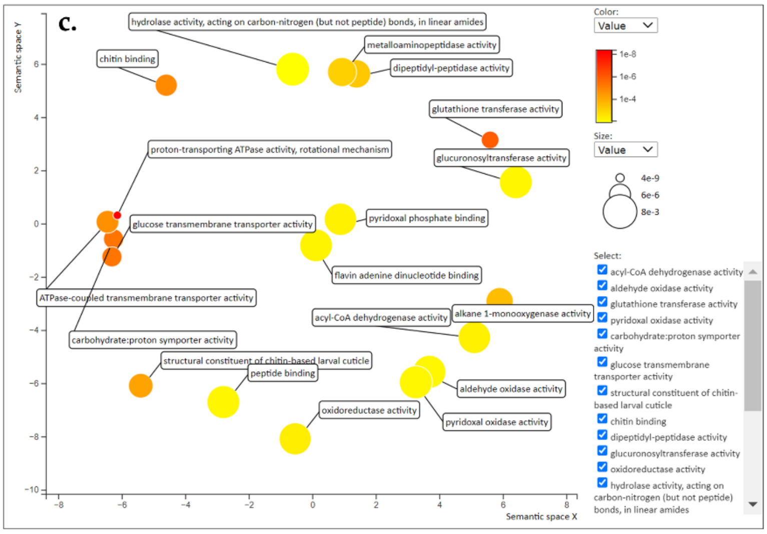

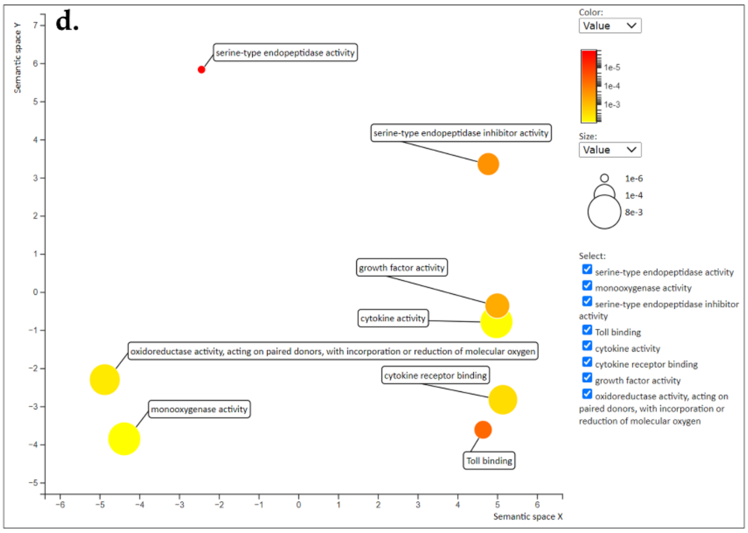

Term ID	Name	Frequency	Pin?	Value	Uniqueness	Dispensability
GO:0008010	structural constituent of chitin-based larval cuticle	0.002%		0.0000	1.00	0.00
GO:0016811	hydrolase activity, acting on carbon-nitrogen (but not peptide) bonds, in linear amides	0.737%		0.0084	0.93	0.00
GO:0042277	peptide binding	0.100%		0.0051	0.98	0.00
GO:0042626	ATPase-coupled transmembrane transporter activity	1.389%		0.0000	0.83	0.00
GO:0004732	pyridoxal oxidase activity	0.000%		0.0055	0.87	0.02
GO:0015020	glucuronosyltransferase activity	0.043%		0.0051	0.96	0.03
GO:0016491	oxidoreductase activity	12.887%		0.0032	0.97	0.05
GO:0050660	flavin adenine dinucleotide binding	1.631%		0.0044	0.96	0.05
GO:0008061	chitin binding	0.116%		0.0000	0.98	0.05
GO:0004364	glutathione transferase activity	0.086%		0.0000	0.96	0.16
GO:0003995	acyl-CoA dehydrogenase activity	0.152%		0.0038	0.92	0.16
GO:0018685	alkane 1-monooxygenase activity	0.001%		0.0002	0.93	0.20
GO:0070006	metalloaminopeptidase activity	0.153%		0.0006	0.89	0.23
GO:0030170	pyridoxal phosphate binding	1.138%		0.0038	0.96	0.31
GO:0005355	glucose transmembrane transporter activity	0.012%		0.0000	0.86	0.33
GO:0008239	dipeptidyl-peptidase activity	0.046%		0.0004	0.89	0.65
GO:0005351	carbohydrate:proton symporter activity	0.026%		0.0000	0.80	0.71
GO:0046961	proton-transporting ATPase activity, rotational mechanism	0.115%		0.0000	0.83	0.71
GO:0004031	aldehyde oxidase activity	0.002%		0.0055	0.86	0.80

Term ID	Name	Frequency	Pin?	Value	Uniqueness	Dispensability
GO:0004497	monooxygenase activity	1.211%		0.0081	0.88	0.00
GO:0005125	cytokine activity	0.080%		0.0079	0.38	0.00
GO:0004252	serine-type endopeptidase activity	0.893%		0.0000	0.98	0.04
GO:0016705	oxidoreductase activity, acting on paired donors, with incorporation or reduction of molecular oxygen	1.285%		0.0040	0.88	0.41
GO:0005121	Toll binding	0.000%		0.0000	0.59	0.53
GO:0004867	serine-type endopeptidase inhibitor activity	0.121%		0.0002	0.69	0.58
GO:0005126	cytokine receptor binding	0.094%		0.0025	0.50	0.74
GO:0008083	growth factor activity	0.106%		0.0005	0.37	0.85

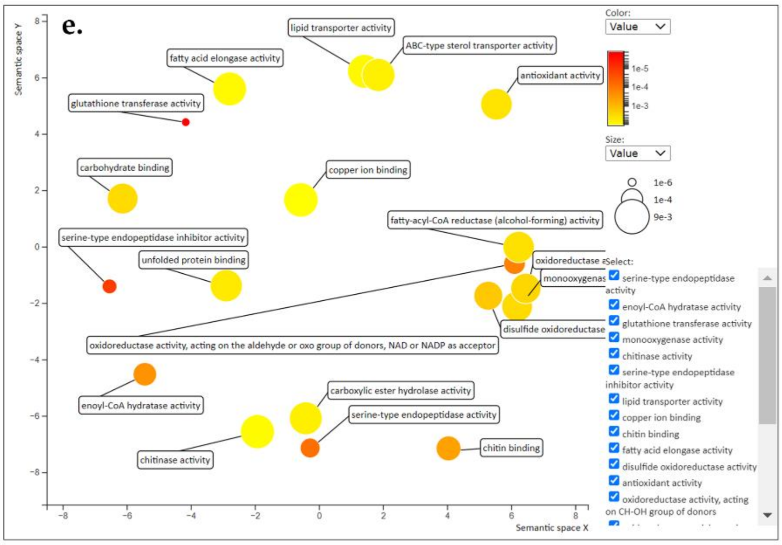

Term ID	Name	Frequency	Pin?	Value	Uniqueness	Dispensability
GO:0004568	chitinase activity	0.057%		0.0084	0.93	0.00
GO:0004867	serine-type endopeptidase inhibitor activity	0.121%		0.0000	1.00	0.00
GO:0005319	lipid transporter activity	0.146%		0.0076	0.94	0.00
GO:0005507	copper ion binding	0.292%		0.0093	0.99	0.00
GO:0016209	antioxidant activity	0.725%		0.0036	0.91	0.00
GO:0004601	peroxidase activity	0.427%		0.0008	0.73	0.91
GO:0080019	fatty-acyl-CoA reductase (alcohol-forming) activity	0.018%		0.0033	0.81	0.02
GO:0009922	fatty acid elongase activity	0.028%		0.0078	0.96	0.02
GO:0004300	enoyl-CoA hydratase activity	0.051%		0.0002	0.97	0.02
GO:0008061	chitin binding	0.116%		0.0004	0.99	0.04
GO:0051082	unfolded protein binding	0.424%		0.0045	0.98	0.05
GO:0030246	carbohydrate binding	0.797%		0.0027	0.98	0.05
GO:0004364	glutathione transferase activity	0.086%		0.0000	0.96	0.16
GO:0052689	carboxylic ester hydrolase activity	0.694%		0.0055	0.92	0.21
GO:0004252	serine-type endopeptidase activity	0.893%		0.0001	0.92	0.27
GO:0004497	monooxygenase activity	1.211%		0.0026	0.80	0.27
GO:0015036	disulfide oxidoreductase activity	0.326%		0.0014	0.82	0.35
GO:0016614	oxidoreductase activity, acting on CH-OH group of donors	2.116%		0.0023	0.79	0.43
GO:0034041	ABC-type sterol transporter activity	0.000%		0.0058	0.94	0.57
GO:0016620	oxidoreductase activity, acting on the aldehyde or oxo group of donors, NAD or NADP as acceptor	0.593%		0.0001	0.77	0.70

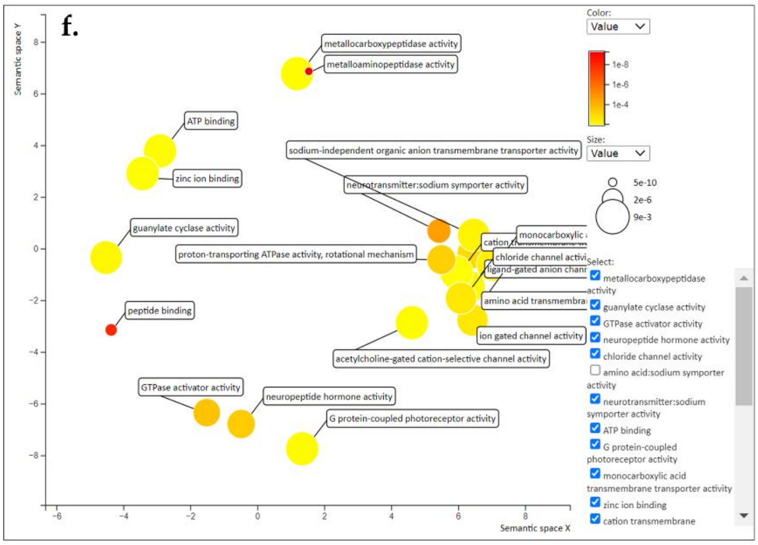

Term ID	Name	Frequency	Pin?	Value	Uniqueness	Dispensability
GO:0004181	metallocarboxypeptidase activity	0.086%		0.0081	0.90	0.00
GO:0008270	zinc ion binding	3.638%		0.0092	0.96	0.00
GO:0008324	cation transmembrane transporter activity	2.405%		0.0090	0.53	0.00
GO:0008020	G protein-coupled photoreceptor activity	0.003%		0.0078	0.91	0.01
GO:0042277	peptide binding	0.100%		0.0000	0.98	0.05
GO:0004383	guanylate cyclase activity	0.024%		0.0059	0.97	0.10
GO:0005184	neuropeptide hormone activity	0.013%		0.0003	0.87	0.25
GO:0005524	ATP binding	13.644%		0.0081	0.96	0.27
GO:0005328	neurotransmitter:sodium symporter activity	0.001%		0.0000	0.63	0.43
GO:0015347	sodium-independent organic anion transmembrane transporter activity	0.001%		0.0048	0.60	0.44
GO:0005096	GTPase activator activity	0.247%		0.0002	0.91	0.54
GO:0022848	acetylcholine-gated cation-selective channel activity	0.024%		0.0078	0.49	0.56
GO:0008028	monocarboxylic acid transmembrane transporter activity	0.052%		0.0042	0.54	0.57
GO:0005254	chloride channel activity	0.123%		0.0073	0.51	0.63
GO:0046961	proton-transporting ATPase activity, rotational mechanism	0.115%		0.0005	0.56	0.65
GO:0022839	ion gated channel activity	0.054%		0.0022	0.63	0.65
GO:0005283	amino acid:sodium symporter activity	0.045%		0.0009	0.50	0.74
GO:0099095	ligand-gated anion channel activity	0.006%		0.0022	0.55	0.80
GO:0070006	metalloaminopeptidase activity	0.153%		0.0000	0.90	0.83
GO:0015171	amino acid transmembrane transporter activity	0.268%		0.0001	0.60	0.83

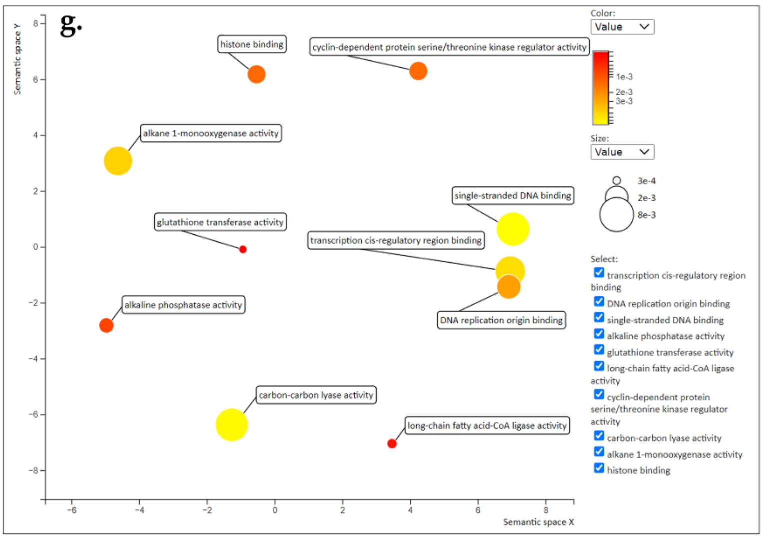

Term ID	Name	Frequency	Pin?	Value	Uniqueness	Dispensability
GO:0003697	single-stranded DNA binding	0.255%		0.0079	0.86	0.00
GO:0016538	cyclin-dependent protein serine/threonine kinase regulator activity	0.069%		0.0012	1.00	0.00
GO:0016830	carbon-carbon lyase activity	1.062%		0.0074	0.98	0.00
GO:0018685	alkane 1-monooxygenase activity	0.001%		0.0045	0.98	0.02
GO:0004035	alkaline phosphatase activity	0.021%		0.0008	0.98	0.03
GO:0004364	glutathione transferase activity	0.086%		0.0003	0.98	0.03
GO:0004467	long-chain fatty acid-CoA ligase activity	0.034%		0.0004	0.98	0.04
GO:0042393	histone binding	0.086%		0.0012	0.97	0.04
GO:0000976	transcription cis-regulatory region binding	0.373%		0.0053	0.75	0.33
GO:0003688	DNA replication origin binding	0.043%		0.0024	0.77	0.81

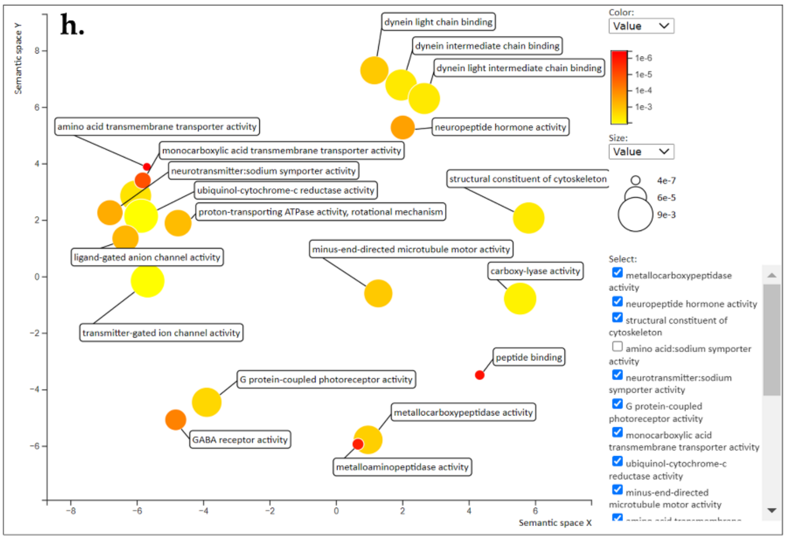

Term ID	Name	Frequency	Pin?	Value	Uniqueness	Dispensability
GO:0008121	ubiquinol-cytochrome-c reductase activity	0.036%		0.0094	0.66	0.00
GO:0008569	minus-end-directed microtubule motor activity	0.040%		0.0011	0.96	0.00
GO:0051959	dynein light intermediate chain binding	0.026%		0.0039	0.90	0.00
GO:0005200	structural constituent of cytoskeleton	0.076%		0.0041	0.99	0.01
GO:0016831	carboxy-lyase activity	0.619%		0.0055	0.99	0.03
GO:0004181	metallocarboxypeptidase activity	0.086%		0.0014	0.91	0.03
GO:0042277	peptide binding	0.100%		0.0000	0.99	0.04
GO:0045503	dynein light chain binding	0.003%		0.0011	0.91	0.31
GO:0005184	neuropeptide hormone activity	0.013%		0.0002	0.84	0.34
GO:0045505	dynein intermediate chain binding	0.030%		0.0039	0.90	0.36
GO:0022824	transmitter-gated ion channel activity	0.062%		0.0092	0.55	0.45
GO:0022848	acetylcholine-gated cation-selective channel activity	0.024%		0.0002	0.54	0.94

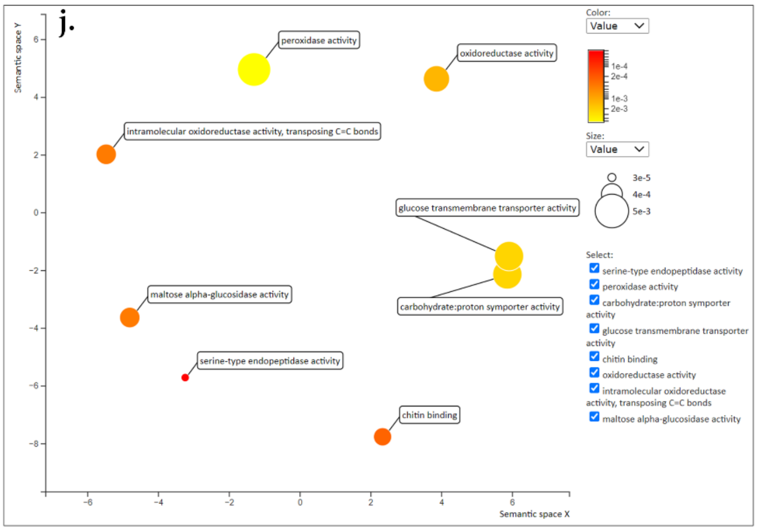

Term ID	Name	Frequency	Pin?	Value	Uniqueness	Dispensability
GO:0004601	peroxidase activity	0.427%		0.0048	0.96	0.00
GO:0008061	chitin binding	0.116%		0.0002	1.00	0.00
GO:0005355	glucose transmembrane transporter activity	0.012%		0.0021	0.80	0.01
GO:0032450	maltose alpha-glucosidase activity	0.006%		0.0004	0.93	0.02
GO:0016863	intramolecular oxidoreductase activity, transposing C=C bonds	0.050%		0.0004	0.97	0.03
GO:0016491	oxidoreductase activity	12.887%		0.0012	0.96	0.04
GO:0004252	serine-type endopeptidase activity	0.893%		0.0000	0.92	0.18
GO:0005351	carbohydrate:proton symporter activity	0.026%		0.0021	0.80	0.71

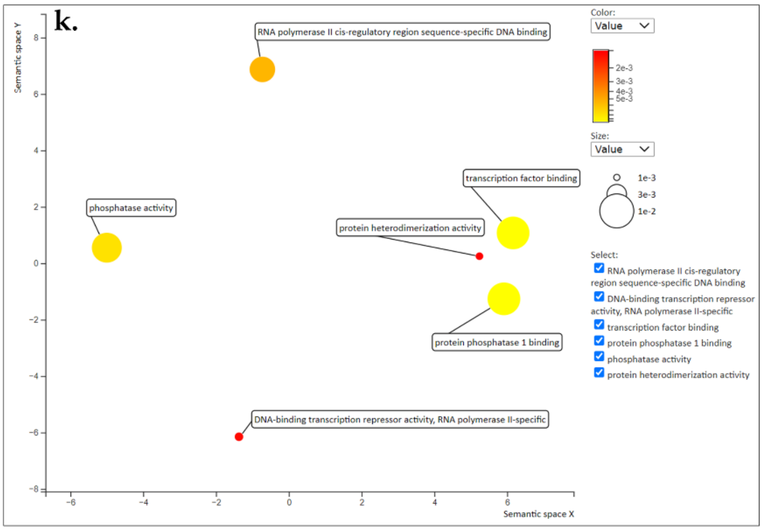

Term ID	Name	Frequency	Pin?	Value	Uniqueness	Dispensability
GO:0001227	DNA-binding transcription repressor activity, RNA polymerase II-specific	0.041%		0.0013	1.00	0.00
GO:0008157	protein phosphatase 1 binding	0.009%		0.0096	0.71	0.00
GO:0016791	phosphatase activity	1.292%		0.0076	1.00	0.00
GO:0000978	RNA polymerase II cis-regulatory region sequence-specific DNA binding	0.193%		0.0053	0.95	0.03
GO:0008134	transcription factor binding	0.111%		0.0096	0.68	0.36
GO:0046982	protein heterodimerization activity	0.275%		0.0012	0.67	0.46

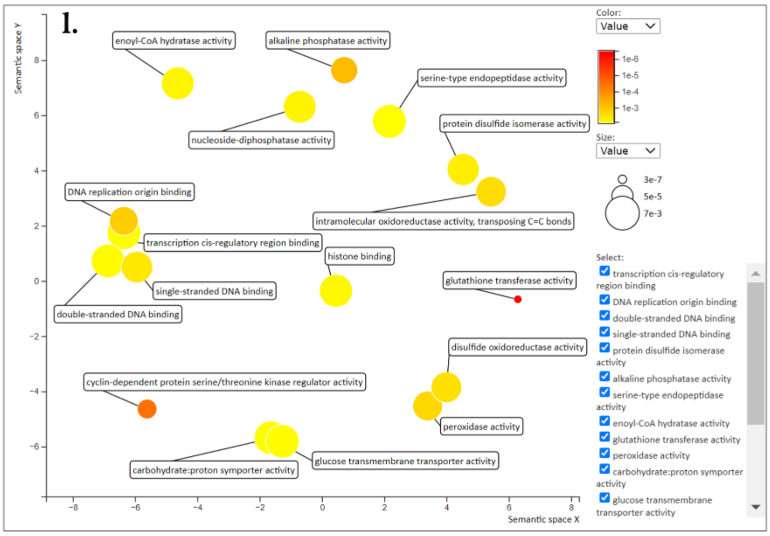

Term ID	Name	Frequency	Pin?	Value	Uniqueness	Dispensability
GO:0003690	double-stranded DNA binding	0.682%		0.0065	0.87	0.00
GO:0004252	serine-type endopeptidase activity	0.893%		0.0074	0.90	0.00
GO:0005355	glucose transmembrane transporter activity	0.012%		0.0066	0.91	0.00
GO:0016538	cyclin-dependent protein serine/threonine kinase regulator activity	0.069%		0.0000	1.00	0.00
GO:0004300	enoyl-CoA hydratase activity	0.051%		0.0051	0.98	0.03
GO:0004364	glutathione transferase activity	0.086%		0.0000	0.98	0.03
GO:0015036	disulfide oxidoreductase activity	0.326%		0.0022	0.94	0.03
GO:0042393	histone binding	0.086%		0.0056	0.98	0.05
GO:0017110	nucleoside-diphosphatase activity	0.016%		0.0047	0.94	0.19
GO:0004035	alkaline phosphatase activity	0.021%		0.0005	0.94	0.20
GO:0003756	protein disulfide isomerase activity	0.028%		0.0038	0.86	0.31
GO:0004601	peroxidase activity	0.427%		0.0015	0.94	0.32
GO:0003697	single-stranded DNA binding	0.255%		0.0028	0.88	0.35
GO:0000976	transcription cis-regulatory region binding	0.373%		0.0071	0.82	0.36
GO:0016863	intramolecular oxidoreductase activity, transposing C=C bonds	0.050%		0.0019	0.90	0.69
GO:0005351	carbohydrate:proton symporter activity	0.026%		0.0066	0.91	0.71
GO:0003688	DNA replication origin binding	0.043%		0.0010	0.83	0.81

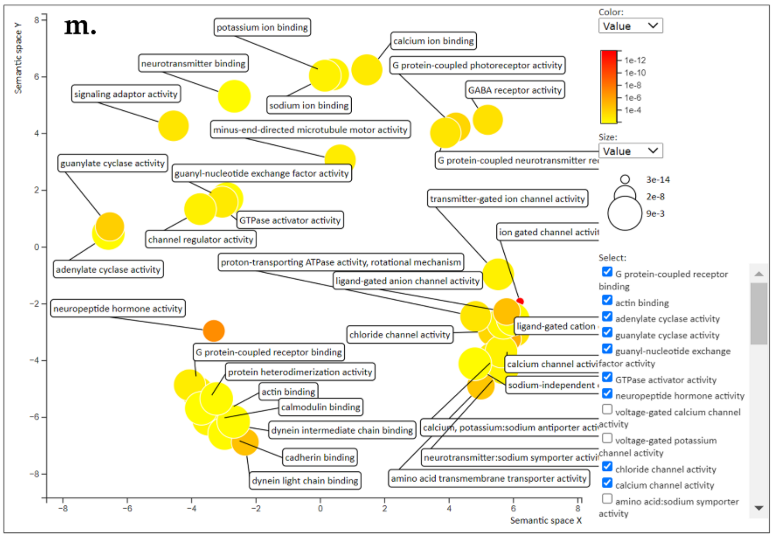

Term ID	Name	Frequency	Pin?	Value	Uniqueness	Dispensability
GO:0003779	actin binding	0.467%		0.0087	0.80	0.00
GO:0004016	adenylate cyclase activity	0.077%		0.0054	0.96	0.00
GO:0005085	guanyl-nucleotide exchange factor activity	0.309%		0.0057	0.88	0.00
GO:0008569	minus-end-directed microtubule motor activity	0.040%		0.0012	0.98	0.00
GO:0015347	sodium-independent organic anion transmembrane transporter activity	0.001%		0.0057	0.72	0.00
GO:0035591	signaling adaptor activity	0.023%		0.0006	1.00	0.00
GO:0042165	neurotransmitter binding	0.001%		0.0057	0.98	0.03
GO:0031402	sodium ion binding	0.009%		0.0021	0.93	0.04
GO:0099528	G protein-coupled neurotransmitter receptor activity	0.007%		0.0006	0.84	0.14
GO:0005509	calcium ion binding	1.117%		0.0010	0.95	0.21
GO:0099094	ligand-gated cation channel activity	0.080%		0.0050	0.58	0.36
GO:0045503	dynein light chain binding	0.003%		0.0000	0.85	0.37
GO:0005328	neurotransmitter:sodium symporter activity	0.001%		0.0025	0.70	0.40
GO:0045296	cadherin binding	0.018%		0.0052	0.83	0.42
GO:0051959	dynein light intermediate chain binding	0.026%		0.0052	0.83	0.43
GO:0045505	dynein intermediate chain binding	0.030%		0.0052	0.83	0.44
GO:0001664	G protein-coupled receptor binding	0.082%		0.0018	0.79	0.47
GO:0008020	G protein-coupled photoreceptor activity	0.003%		0.0001	0.88	0.48
GO:0016917	GABA receptor activity	0.027%		0.0004	0.90	0.48
GO:0005516	calmodulin binding	0.135%		0.0027	0.81	0.49
GO:0046982	protein heterodimerization activity	0.275%		0.0062	0.81	0.52
GO:0046961	proton-transporting ATPase activity, rotational mechanism	0.115%		0.0006	0.64	0.54

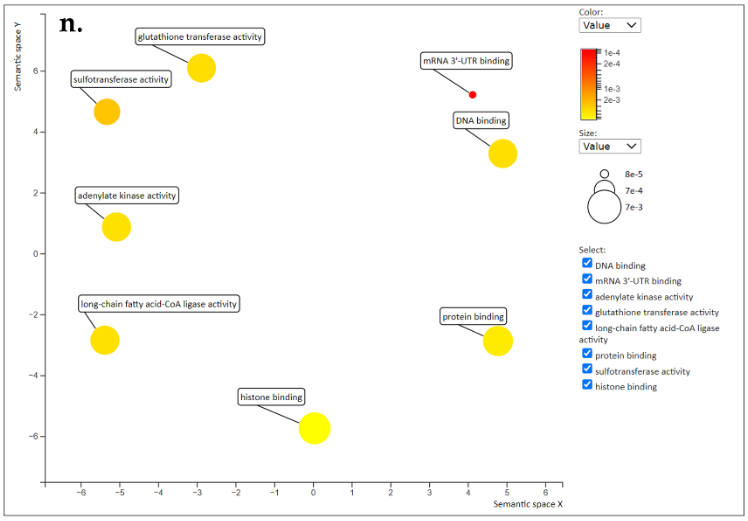

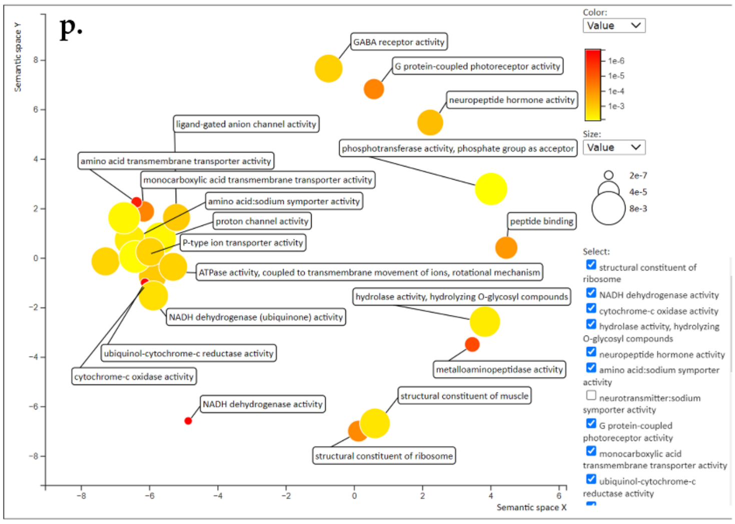

Term ID	Name	Frequency	Pin?	Value	Uniqueness	Dispensability
GO:0004467	long-chain fatty acid-CoA ligase activity	0.034%		0.0039	0.93	0.00
GO:0042393	histone binding	0.086%		0.0066	0.96	0.00
GO:0004364	glutathione transferase activity	0.086%		0.0037	0.90	0.02
GO:0003730	mRNA 3'-UTR binding	0.026%		0.0001	0.91	0.04
GO:0005515	protein binding	4.557%		0.0047	0.94	0.05
GO:0008146	sulfotransferase activity	0.121%		0.0024	0.90	0.17
GO:0004017	adenylate kinase activity	0.059%		0.0039	0.85	0.20
GO:0003677	DNA binding	11.354%		0.0038	0.88	0.26

Term ID	Name	Frequency	Pin?	Value	Uniqueness	Dispensability
GO:0008307	structural constituent of muscle	0.004%		0.0025	0.96	0.00
GO:0015662	P-type ion transporter activity	0.107%		0.0065	0.48	0.00
GO:0016776	phosphotransferase activity, phosphate group as acceptor	0.361%		0.0076	0.99	0.00
GO:0016917	GABA receptor activity	0.027%		0.0011	0.96	0.00
GO:0005184	neuropeptide hormone activity	0.013%		0.0005	0.97	0.01
GO:0004553	hydrolase activity, hydrolyzing O-glycosyl compounds	1.613%		0.0033	0.97	0.03
GO:0042277	peptide binding	0.100%		0.0001	1.00	0.03
GO:0070006	metalloaminopeptidase activity	0.153%		0.0000	0.97	0.24
GO:0015217	ADP transmembrane transporter activity	0.020%		0.0051	0.57	0.43
GO:0008020	G protein-coupled photoreceptor activity	0.003%		0.0000	0.93	0.46
GO:0003735	structural constituent of ribosome	2.348%		0.0001	0.96	0.50
GO:0015252	proton channel activity	0.315%		0.0051	0.49	0.52
GO:0099095	ligand-gated anion channel activity	0.006%		0.0008	0.60	0.58
GO:0005283	amino acid:sodium symporter activity	0.045%		0.0034	0.44	0.61
GO:0008137	NADH dehydrogenase (ubiquinone) activity	0.260%		0.0021	0.45	0.61
GO:0044769	ATPase activity, coupled to transmembrane movement of ions, rotational mechanism	0.115%		0.0012	0.52	0.62
GO:0046961	proton-transporting ATPase activity, rotational mechanism	0.115%		0.0003	0.44	1.00

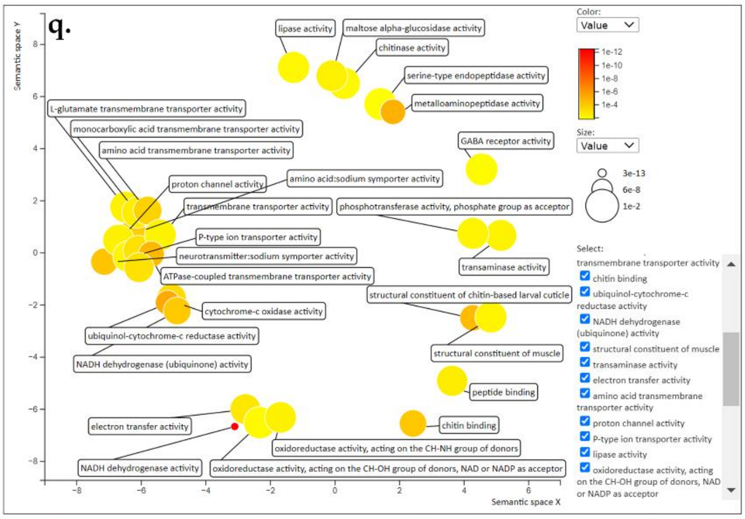

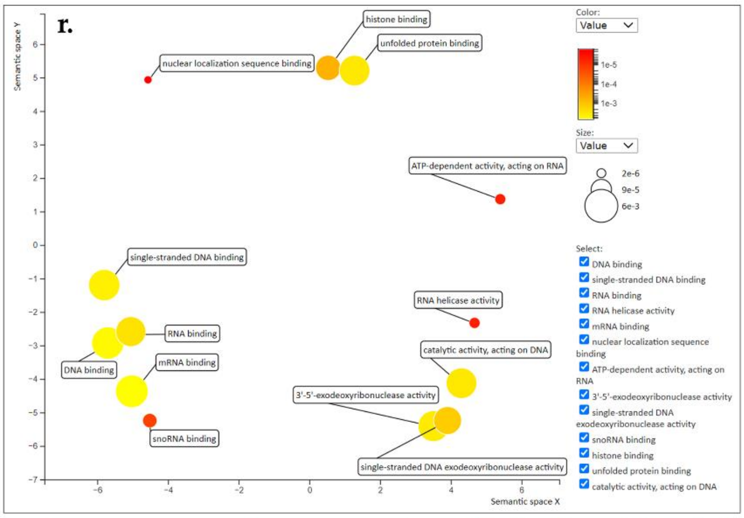

Term ID	Name	Frequency	Pin?	Value	Uniqueness	Dispensability
GO:0004252	serine-type endopeptidase activity	0.893%		0.0096	0.90	0.00
GO:0008307	structural constituent of muscle	0.004%		0.0039	0.98	0.00
GO:0015662	P-type ion transporter activity	0.107%		0.0040	0.55	0.00
GO:0016917	GABA receptor activity	0.027%		0.0073	1.00	0.00
GO:0042277	peptide binding	0.100%		0.0018	1.00	0.00
GO:0016776	phosphotransferase activity, phosphate group as acceptor	0.361%		0.0037	0.96	0.03
GO:0016616	oxidoreductase activity, acting on the CH-OH group of donors, NAD or NADP as acceptor	1.855%		0.0061	0.85	0.04
GO:0008061	chitin binding	0.116%		0.0001	1.00	0.04
GO:0004568	chitinase activity	0.057%		0.0058	0.92	0.21
GO:0008483	transaminase activity	0.728%		0.0036	0.96	0.23
GO:0016298	lipase activity	0.261%		0.0049	0.93	0.24
GO:0008010	structural constituent of chitin-based larval cuticle	0.002%		0.0000	0.98	0.33
GO:0016645	oxidoreductase activity, acting on the CH-NH group of donors	0.350%		0.0028	0.86	0.37
GO:0005313	L-glutamate transmembrane transporter activity	0.004%		0.0028	0.65	0.39
GO:0009055	electron transfer activity	1.194%		0.0011	0.77	0.42
GO:0032450	maltose alpha-glucosidase activity	0.006%		0.0028	0.91	0.47
GO:0005328	neurotransmitter:sodium symporter activity	0.001%		0.0000	0.65	0.47
GO:0015252	proton channel activity	0.315%		0.0028	0.57	0.52
GO:0022857	transmembrane transporter activity	9.001%		0.0033	0.61	0.53
GO:0070006	metalloaminopeptidase activity	0.153%		0.0000	0.91	0.54

Term ID	Name	Frequency	Pin?	Value	Uniqueness	Dispensability
GO:0003729	mRNA binding	0.276%		0.0056	0.78	0.00
GO:0008186	ATP-dependent activity, acting on RNA	0.334%		0.0000	0.92	0.00
GO:0140097	catalytic activity, acting on DNA	2.626%		0.0028	0.79	0.00
GO:0008139	nuclear localization sequence binding	0.010%		0.0000	0.96	0.04
GO:0051082	unfolded protein binding	0.424%		0.0028	0.87	0.05
GO:0003697	single-stranded DNA binding	0.255%		0.0035	0.81	0.22
GO:0003677	DNA binding	11.354%		0.0048	0.75	0.33
GO:0008296	3'-5'-exodeoxyribonuclease activity	0.010%		0.0031	0.78	0.39
GO:0030515	snoRNA binding	0.048%		0.0000	0.80	0.40
GO:0042393	histone binding	0.086%		0.0005	0.88	0.47
GO:0003723	RNA binding	5.630%		0.0023	0.76	0.53
GO:0003724	RNA helicase activity	0.307%		0.0000	0.73	0.54
GO:0008297	single-stranded DNA exodeoxyribonuclease activity	0.006%		0.0011	0.78	0.70

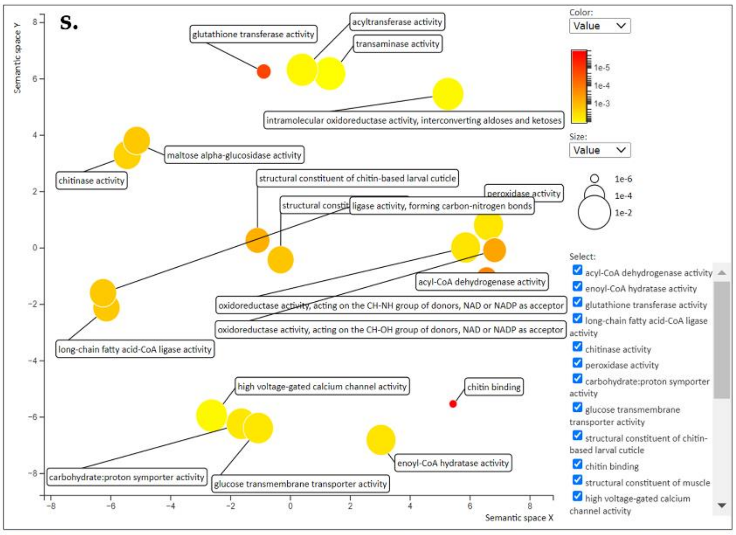

Term ID	Name	Frequency	Pin?	Value	Uniqueness	Dispensability
GO:0008061	chitin binding	0.116%		0.0000	1.00	0.00
GO:0008307	structural constituent of muscle	0.004%		0.0013	0.96	0.00
GO:0008331	high voltage-gated calcium channel activity	0.001%		0.0081	0.93	0.00
GO:0008483	transaminase activity	0.728%		0.0100	0.92	0.00
GO:0004300	enoyl-CoA hydratase activity	0.051%		0.0039	0.97	0.03
GO:0004568	chitinase activity	0.057%		0.0020	0.92	0.03
GO:0016861	intramolecular oxidoreductase activity, interconverting aldoses and ketoses	0.309%		0.0081	0.96	0.03
GO:0004601	peroxidase activity	0.427%		0.0042	0.86	0.03
GO:0016879	ligase activity, forming carbon-nitrogen bonds	1.271%		0.0015	0.90	0.04
GO:0004364	glutathione transferase activity	0.086%		0.0000	0.93	0.20
GO:0005355	glucose transmembrane transporter activity	0.012%		0.0044	0.90	0.22
GO:0016746	acyltransferase activity	3.062%		0.0079	0.91	0.29
GO:0003995	acyl-CoA dehydrogenase activity	0.152%		0.0001	0.87	0.30
GO:0016646	oxidoreductase activity, acting on the CH-NH group of donors, NAD or NADP as acceptor	0.269%		0.0039	0.87	0.31
GO:0008010	structural constituent of chitin-based larval cuticle	0.002%		0.0006	0.96	0.33
GO:0016616	oxidoreductase activity, acting on the CH-OH group of donors, NAD or NADP as acceptor	1.855%		0.0004	0.85	0.38

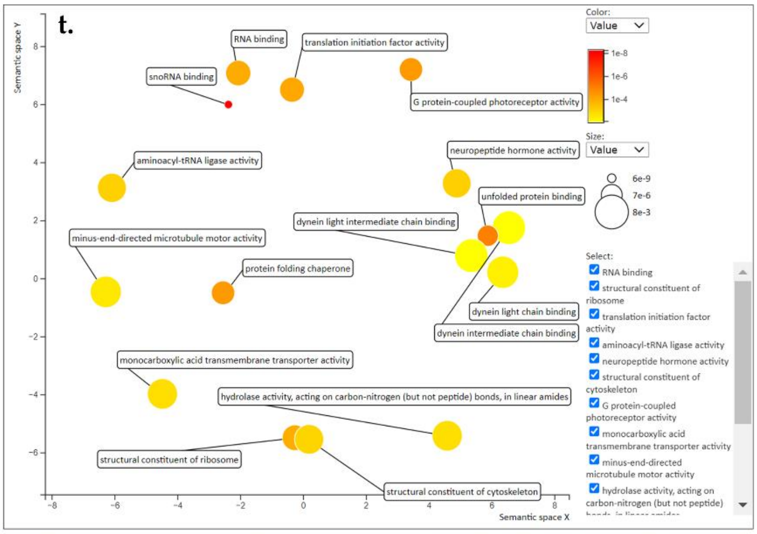

Term ID	Name	Frequency	Pin?	Value	Uniqueness	Dispensability
GO:0008028	monocarboxylic acid transmembrane transporter activity	0.052%		0.0014	1.00	0.00
GO:0008569	minus-end-directed microtubule motor activity	0.040%		0.0023	1.00	0.00
GO:0016811	hydrolase activity, acting on carbon-nitrogen (but not peptide) bonds, in linear amides	0.737%		0.0014	1.00	0.00
GO:0051959	dynein light intermediate chain binding	0.026%		0.0080	0.80	0.00
GO:0044183	protein folding chaperone	0.019%		0.0000	1.00	0.01
GO:0005200	structural constituent of cytoskeleton	0.076%		0.0008	0.91	0.01
GO:0004812	aminoacyl-tRNA ligase activity	0.895%		0.0006	1.00	0.03
GO:0003743	translation initiation factor activity	0.394%		0.0001	0.84	0.04
GO:0008020	G protein-coupled photoreceptor activity	0.003%		0.0000	0.94	0.16
GO:0045503	dynein light chain binding	0.003%		0.0037	0.82	0.31
GO:0003723	RNA binding	5.630%		0.0001	0.89	0.32
GO:0005184	neuropeptide hormone activity	0.013%		0.0006	0.76	0.34
GO:0045505	dynein intermediate chain binding	0.030%		0.0080	0.80	0.36
GO:0030515	snoRNA binding	0.048%		0.0000	0.89	0.41
GO:0051082	unfolded protein binding	0.424%		0.0000	0.77	0.43
GO:0003735	structural constituent of ribosome	2.348%		0.0001	0.92	0.63

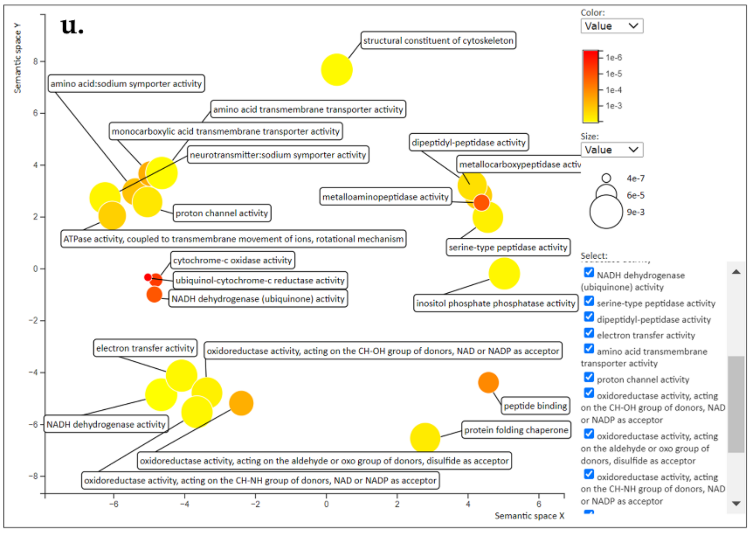

Term ID	Name	Frequency	Pin?	Value	Uniqueness	Dispensability
GO:0003954	NADH dehydrogenase activity	0.374%		0.0094	0.76	0.00
GO:0015171	amino acid transmembrane transporter activity	0.268%		0.0079	0.68	0.00
GO:0042277	peptide binding	0.100%		0.0001	1.00	0.00
GO:0044183	protein folding chaperone	0.019%		0.0043	0.99	0.01
GO:0005200	structural constituent of cytoskeleton	0.076%		0.0076	0.99	0.01
GO:0052745	inositol phosphate phosphatase activity	0.046%		0.0067	0.89	0.02
GO:0008236	serine-type peptidase activity	1.328%		0.0051	0.75	0.21
GO:0004252	*serine-type endopeptidase activity*	*0.893%*		*0.0001*	*0.76*	*0.94*
GO:0005328	neurotransmitter:sodium symporter activity	0.001%		0.0064	0.69	0.25
GO:0016624	oxidoreductase activity, acting on the aldehyde or oxo group of donors, disulfide as acceptor	0.123%		0.0004	0.82	0.29
GO:0016646	oxidoreductase activity, acting on the CH-NH group of donors, NAD or NADP as acceptor	0.269%		0.0067	0.81	0.31
GO:0009055	electron transfer activity	1.194%		0.0071	0.69	0.36
GO:0044769	ATPase activity, coupled to transmembrane movement of ions, rotational mechanism	0.115%		0.0015	0.64	0.37
GO:0046961	*proton-transporting ATPase activity, rotational mechanism*	*0.115%*		*0.0001*	*0.57*	*1.00*
GO:0016616	oxidoreductase activity, acting on the CH-OH group of donors, NAD or NADP as acceptor	1.855%		0.0053	0.78	0.42

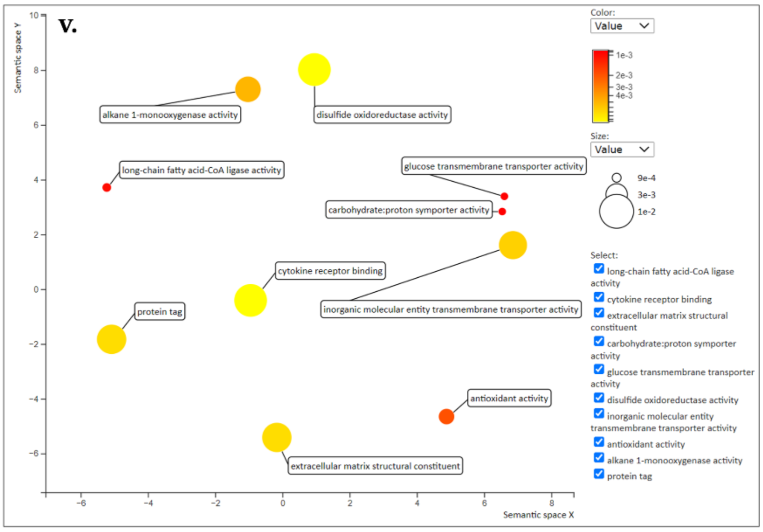

Term ID	Name	Frequency	Pin?	Value	Uniqueness	Dispensability
GO:0005126	cytokine receptor binding	0.094%		0.0096	1.00	0.00
GO:0005201	extracellular matrix structural constituent	0.037%		0.0070	1.00	0.00
GO:0015036	disulfide oxidoreductase activity	0.326%		0.0096	0.95	0.00
GO:0015318	inorganic molecular entity transmembrane transporter activity	2.924%		0.0062	0.84	0.00
GO:0031386	protein tag	0.011%		0.0070	1.00	0.00
GO:0016209	antioxidant activity	0.725%		0.0019	0.99	0.01
GO:0004467	long-chain fatty acid-CoA ligase activity	0.034%		0.0010	0.98	0.02
GO:0018685	alkane 1-monooxygenase activity	0.001%		0.0048	0.95	0.21
GO:0005355	glucose transmembrane transporter activity	0.012%		0.0009	0.78	0.35
GO:0005351	carbohydrate:proton symporter activity	0.026%		0.0009	0.77	0.71

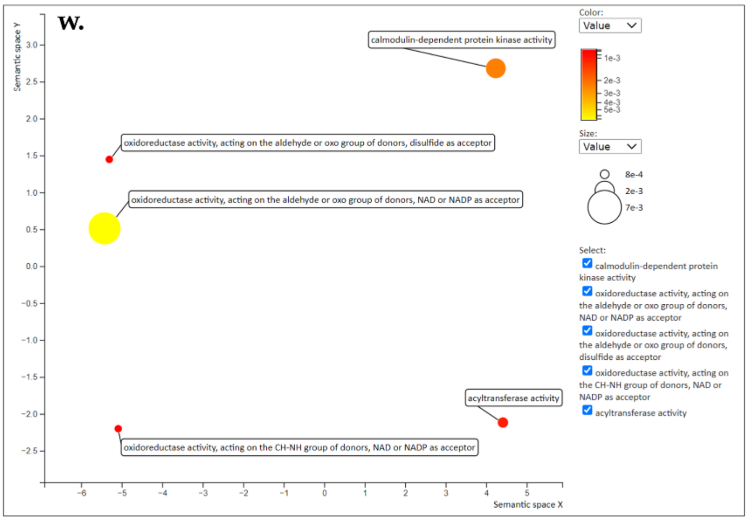

Term ID	Name	Frequency	Pin?	Value	Uniqueness	Dispensability
GO:0016620	oxidoreductase activity, acting on the aldehyde or oxo group of donors, NAD or NADP as acceptor	0.593%		0.0067	0.49	0.00
GO:0004683	calmodulin-dependent protein kinase activity	0.022%		0.0023	0.87	0.02
GO:0016746	acyltransferase activity	3.062%		0.0010	0.85	0.20
GO:0016646	oxidoreductase activity, acting on the CH-NH group of donors, NAD or NADP as acceptor	0.269%		0.0008	0.70	0.32
GO:0016624	oxidoreductase activity, acting on the aldehyde or oxo group of donors, disulfide as acceptor	0.123%		0.0008	0.51	0.81

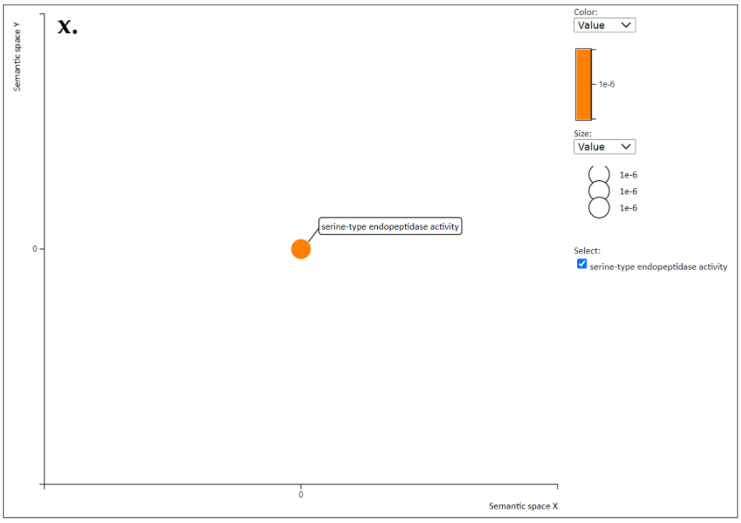

Term ID	Name	Frequency	Pin?	Value	Uniqueness	Dispensability
GO:0004252	serine-type endopeptidase activity	0.893%		0.0000	1.00	0.00

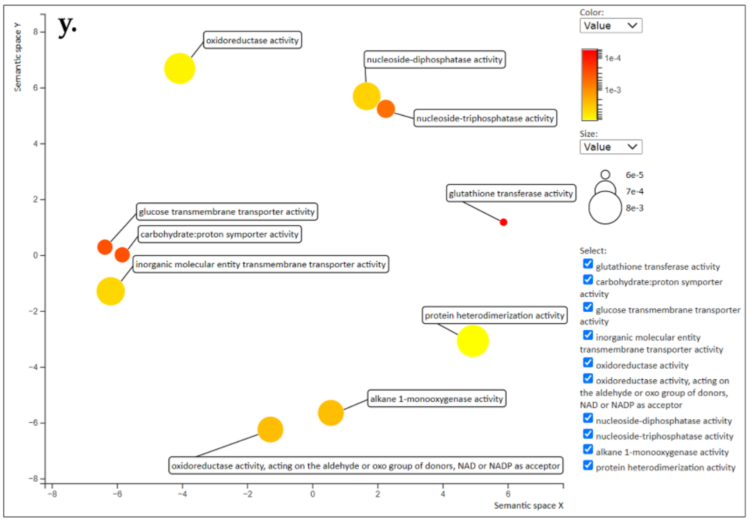

Term ID	Name	Frequency	Pin?	Value	Uniqueness	Dispensability
GO:0015318	inorganic molecular entity transmembrane transporter activity	2.924%		0.0038	0.85	0.00
GO:0017110	nucleoside-diphosphatase activity	0.016%		0.0034	0.87	0.00
GO:0046982	protein heterodimerization activity	0.275%		0.0083	1.00	0.00
GO:0018685	alkane 1-monooxygenase activity	0.001%		0.0023	0.93	0.02
GO:0004364	glutathione transferase activity	0.086%		0.0001	0.97	0.02
GO:0016491	oxidoreductase activity	12.887%		0.0067	0.96	0.04
GO:0016620	oxidoreductase activity, acting on the aldehyde or oxo group of donors, NAD or NADP as acceptor	0.593%		0.0023	0.92	0.22
GO:0005355	glucose transmembrane transporter activity	0.012%		0.0003	0.78	0.35
GO:0017111	nucleoside-triphosphatase activity	3.353%		0.0005	0.86	0.52
GO:0005351	carbohydrate:proton symporter activity	0.026%		0.0003	0.77	0.71

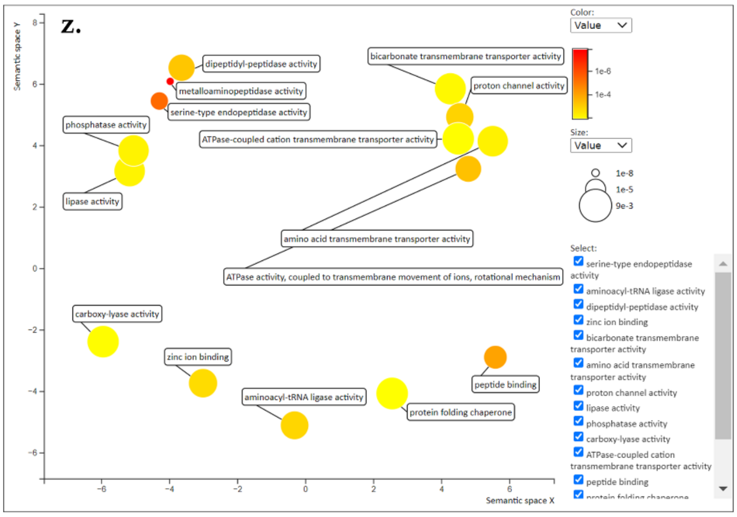

Term ID	Name	Frequency	Pin?	Value	Uniqueness	Dispensability
GO:0008270	zinc ion binding	3.638%		0.0015	0.99	0.00
GO:0016831	carboxy-lyase activity	0.619%		0.0080	0.97	0.00
GO:0044183	protein folding chaperone	0.019%		0.0091	0.99	0.00
GO:0019829	ATPase-coupled cation transmembrane transporter activity	0.352%		0.0088	0.72	0.01
GO:0016298	lipase activity	0.261%		0.0051	0.83	0.03
GO:0004812	aminoacyl-tRNA ligase activity	0.895%		0.0011	0.97	0.03
GO:0042277	peptide binding	0.100%		0.0001	0.99	0.05
GO:0008239	dipeptidyl-peptidase activity	0.046%		0.0005	0.79	0.19
GO:0015171	amino acid transmembrane transporter activity	0.268%		0.0047	0.80	0.38
GO:0015106	bicarbonate transmembrane transporter activity	0.003%		0.0066	0.81	0.41
GO:0004252	serine-type endopeptidase activity	0.893%		0.0000	0.78	0.49
GO:0016791	phosphatase activity	1.292%		0.0048	0.81	0.55
GO:0070006	metalloaminopeptidase activity	0.153%		0.0000	0.77	0.65
GO:0015252	proton channel activity	0.315%		0.0009	0.76	0.66
GO:0044769	ATPase activity, coupled to transmembrane movement of ions, rotational mechanism	0.115%		0.0004	0.78	0.68

Fig. S6: Revigo Scatter and Table Views of the GO terms over-represented in all comparisons (R vs S, R vs C and C vs S) for data from Nigeria, Niger, Chad and Cameroon.

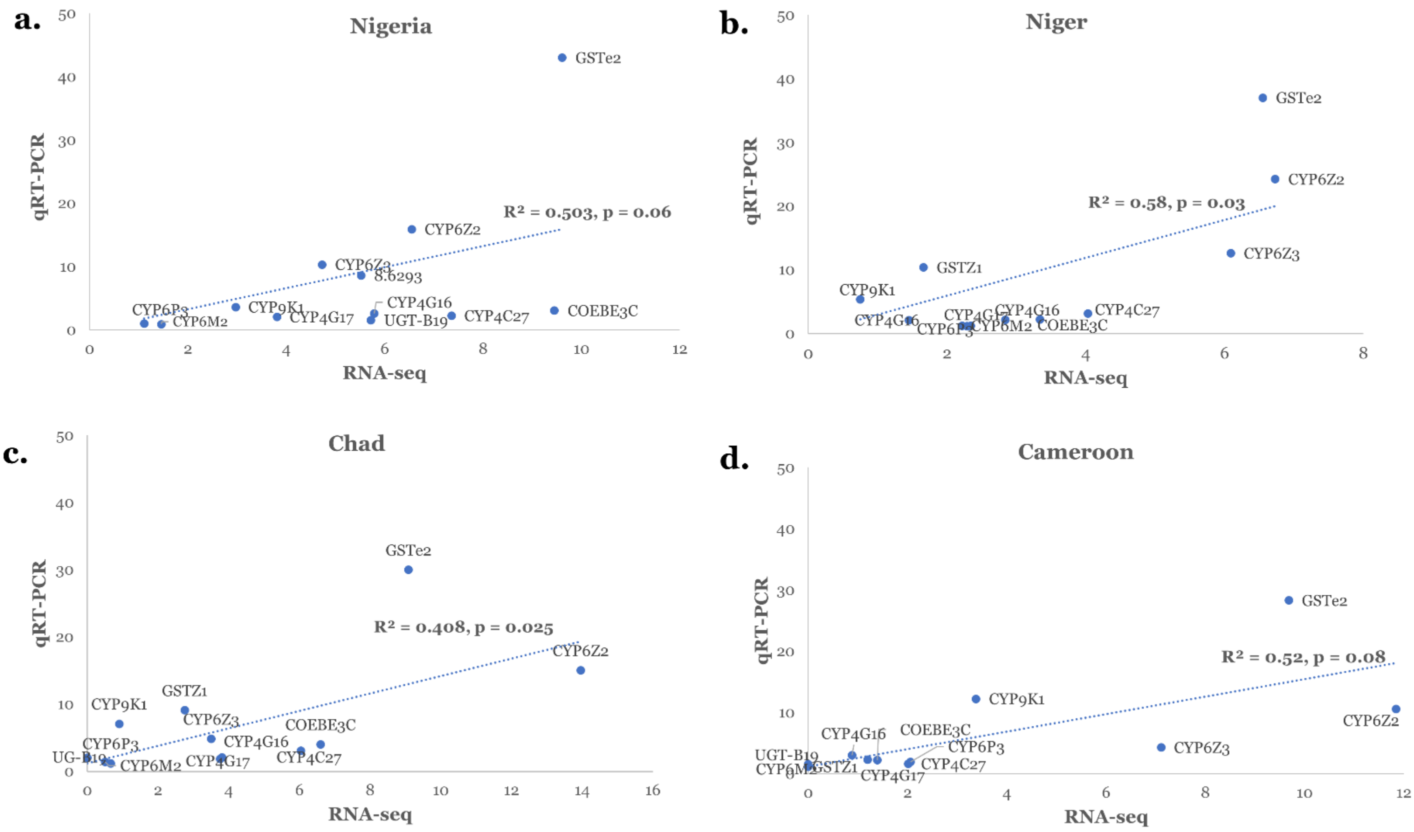

Fig. S7: Validation of RNA-seq results using qRT-PCR: correlation between RNA-seq fold changes and those obtained from qRT-PCR for 12 metabolic resistance genes.

a.

Population	n	S	Syn	Nonsyn	h	H_d	π (k)	D (Tajima)	D* (Fu and Li)
**Nigeria**	4	2	2	0	2	0.667	0.00090	1.893^{ns}	1.893^{ns}
**Niger**	4	42	36	6	3	0.833	0.01848	2.006^{ns}	2.006^{ns}
**Chad**	4	0	0	0	1	0.00	0.0000	0.000	0.000
**Cameroon**	4	23	20	3	2	0.500	0.0078	-0.857^{ns}	0.857^{ns}
**Ngoussou**	4	1	1	0	2	0.500	0.00034	-0.612^{ns}	-0.612^{ns}
**All**	20	75	65	12	10	0.921	0.01648	0.501^{ns}	1.298^{ns}

b.

**Hap #**	**Freq.**	**Sequences**
Hap_1:	2	>Nigeria2 >Nigeria3
Hap_2:	2	>Nigeria1 >Nigeria7
Hap_3:	1	>Niger1
Hap_4:	1	>Niger2
Hap_5:	2	>Niger3 >Niger4
Hap_6:	4	>Chad1 >Chad2 >Chad3 >Chad6
Hap_7:	3	>Cameroon1 >Cameroon2 >Cameroon3
Hap_8:	1	>Cameroon4
Hap_9:	3	>Ngoussou2 >Ngoussou4 >Ngoussou6
Hap_10:	1	>Ngoussou3

c.

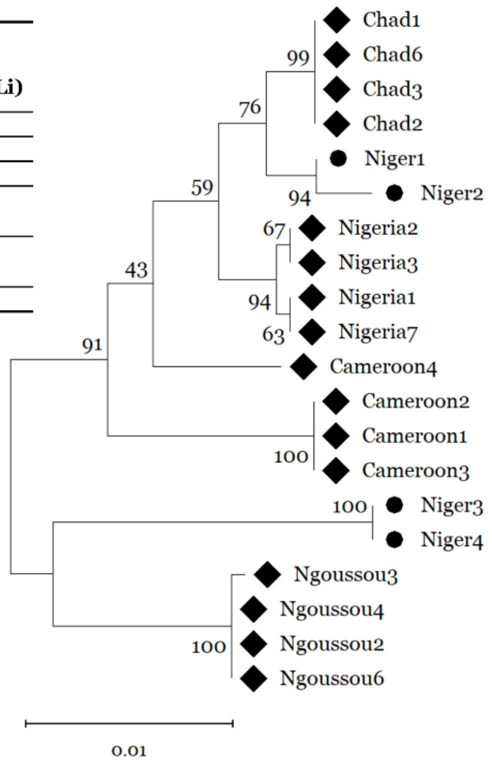

Fig. S8: Polymorphism analysis of full length cDNA of *CYP6Z2*. a. Summary statistics for polymorphisms of *CYP6Z2* cDNA across the Sahel countries. n = number of sequences (n); S, number of polymorphic sites; h, haplotype; H_d, haplotype diversity Syn, Synonymous mutations; Nonsyn, Non-synonymous mutations; π, nucleotide diversity (k= mean number of nucleotide differences); Tajima's D and Fu and Li's D statistics, ns, not significant. **b.** *CYP6Z2* cDNA haplotypes and its frequencies, **c.** a maximum likelihood phylogenetic tree of *CYP6Z2* cDNA sequences.

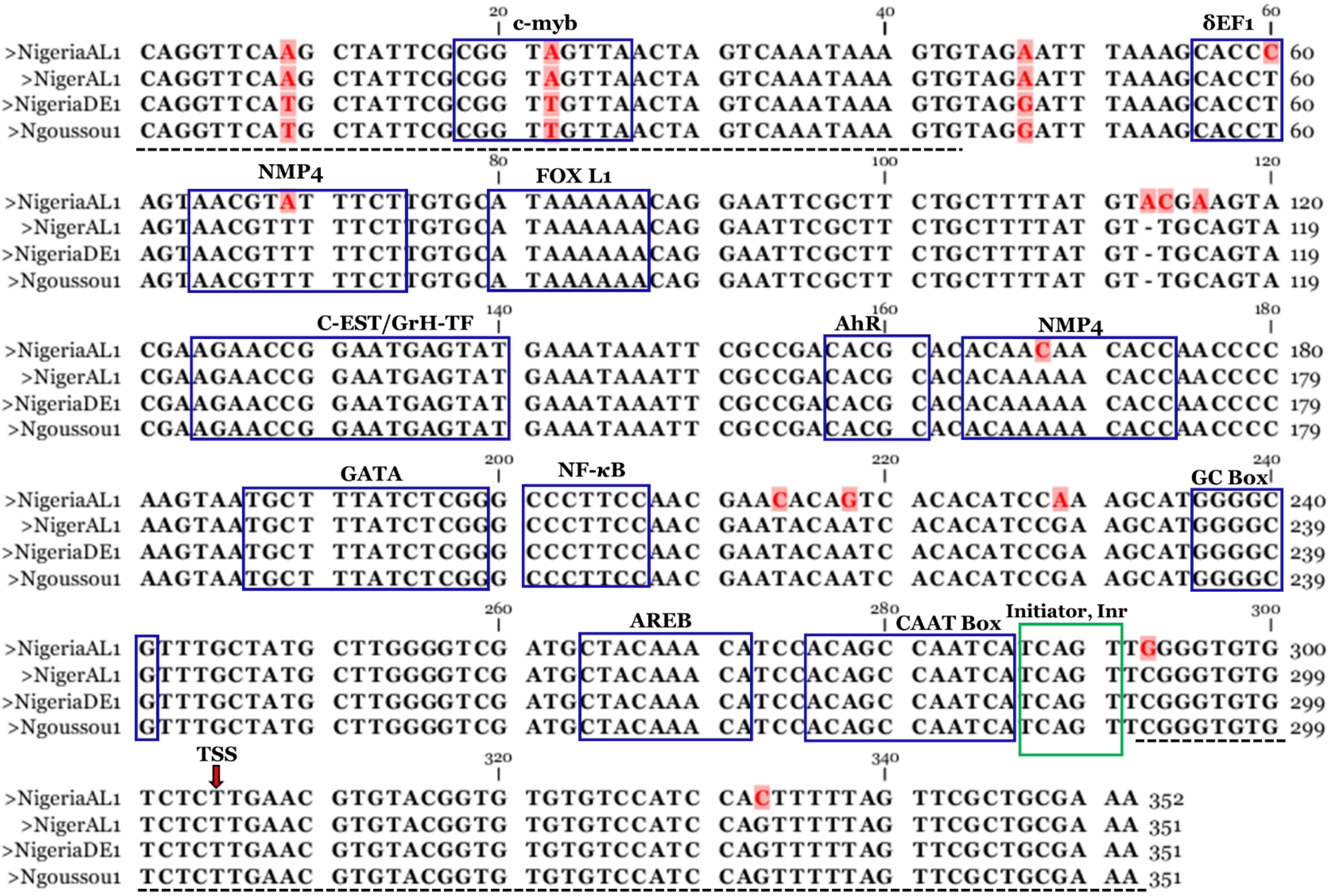

Fig. S9: Comparative alignment of the *GSTe2* 5'-UTR fragments from the various haplotypes, showing putative transcription factors binding sites (blue boxes), and polymorphic positions (in red and highlighted in pink). The arthropod Inr consensus sequence is in green box, the transcription start site indicated with red arrow, and the 3'-UTR of *GSTe1* and 5'-UTR of *GSTe2* indicated with dashed lines.

a.

Hap# Freq. Sequences

Hap_1: 75 NigeriaDE1 NigeriaDE2 NigeriaDE3 NigeriaDE4 NigeriaDE11 NigeriaDE15 NigeriaDE16 NigeriaDE20 NigeriaDE26 Ngoussou1 Ngoussou2 Ngoussou4 Ngoussou5 Ngoussou7 Ngoussou9 Ngoussou11 Ngoussou13 Ngoussou15 Ngoussou16 NigerAL3 NigerAL4 NigerAL5 NigerAL6 NigerAL7 NigerAL8 NigerDE1 NigerDE2 NigerDE3 NigerDE4 NigerDE5 NigerDE6 NigerDE7 NigerDE9 NigerDE11 NigerDE12 ChadAL1 ChadAL3 ChadAL4 ChadAL5 ChadAL6 ChadAL7 ChadAL9 ChadAL10 ChadDE1 ChadDE2 ChadDE3 ChadDE4 ChadDE6 ChadDE7 ChadDE9 ChadDE11 ChadDE13 ChadDE24 CameroonAL1 CameroonAL2 CameroonAL3 CameroonAL4 CameroonAL8 CameroonAL9 CameroonAL11 CameroonDE1 CameroonDE2 CameroonDE3 CameroonDE4 CameroonDE14 CameroonDE12 CameroonDE11 CameroonDE8 CameroonDE10 CameroonDE6 NigeriaAL7 NigeriaAL4 NigeriaAL8 NigeriaAL17 NigeriaAL2

Hap_2: 1 NigeriaDE29

Hap_3: 1 NigeriaAL3

Hap_4: 1 NigeriaAL5

Hap_5: 4 NigerAL1 NigerAL9 NigerAL10 NigerAL11

Hap_6: 8 NigeriaAL1 NigeriaAL6 NigeriaAL9 ChadAL2 ChadAL13 CameroonAL5 CameroonAL25 CameroonAL7

b.

```
       0000001111 2222223
       0246680116 11 23993
       927095246848941 33
Hap_1  TTGTTATTCATAGATCG
Hap_2  ............................G.....
Hap_3  ........... C.....................
Hap_4  ..ACAG. CACCGA .AG.
Hap_5  AAA...........................
Hap_6  AAACA. .CACCGA...GC
```

c.

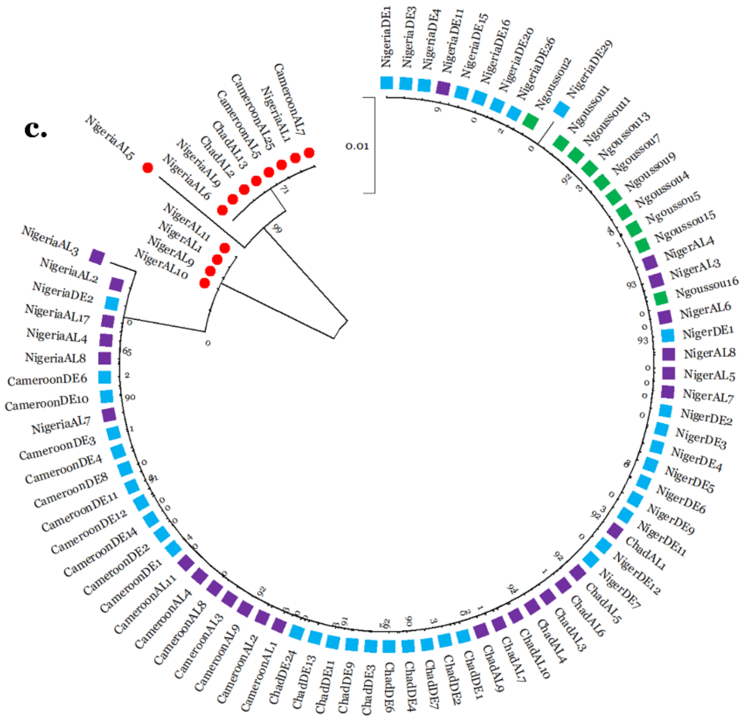

Fig. S10: Genetic variability of the *GSTe2* 5'-UTR fragments from Nigeria, Niger, Chad, Cameroon and Ngoussou populations. a. the six haplotypes of the 5'-UTR with respective frequencies, **b.** polymorphic positions with numbers on top indicating the nucleotide position in the 351 bp fragment, **c.** maximum likelihood phylogenetic tree of the 90 sequences, showing the DDT-alive sequences with mutations forming a clade specific to its phenotype.

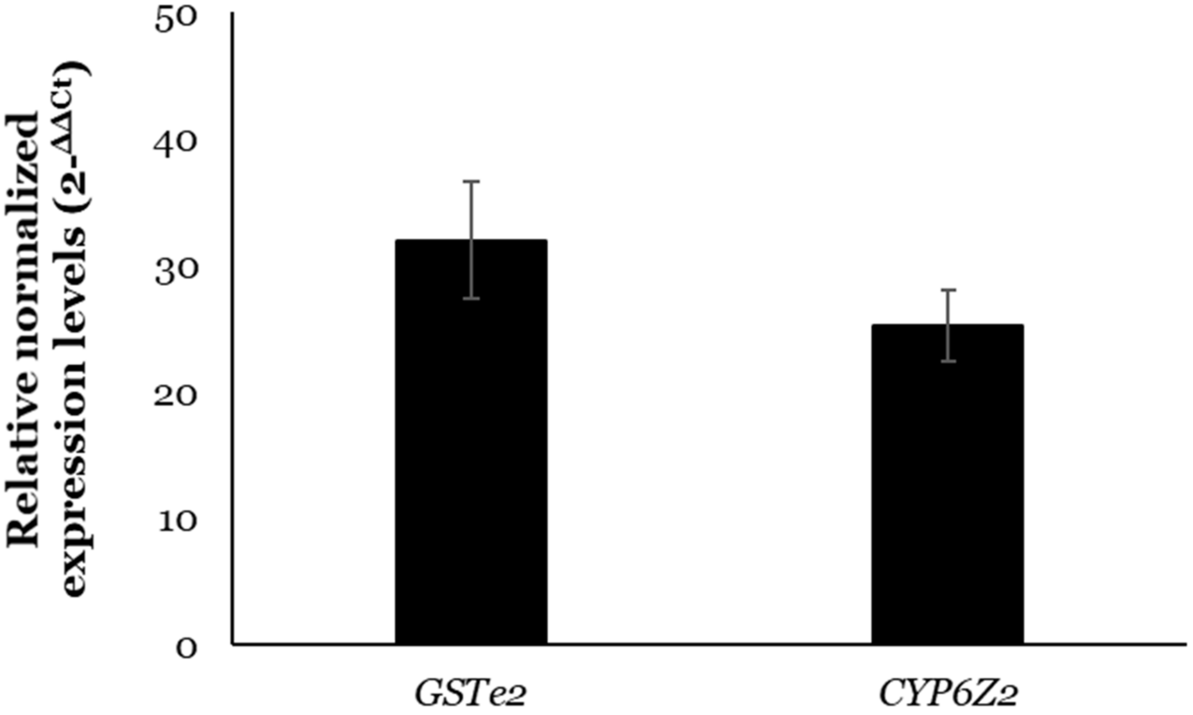

Fig. S11: qRT-PCR validation of the overexpression of *GSTe2* and *CYP6Z2*. The relative expression of the *GSTe2* and *CYP6Z2* in the experimental transgenic *D. melanogaster* in relation to control flies. Data shown are the mean ± standard deviation (n = 6).
